# The Role of Inflammation in Diabetic Retinopathy

**DOI:** 10.3389/fimmu.2020.583687

**Published:** 2020-11-06

**Authors:** John V. Forrester, Lucia Kuffova, Mirela Delibegovic

**Affiliations:** ^1^ Institute of Medical Sciences, University of Aberdeen, Scotland, United Kingdom; ^2^ Eye Clinic, Aberdeen Royal Infirmary, Aberdeen, United Kingdom

**Keywords:** diabetes, retinopathy, inflammation, metabolic syndrome, obesity, protein tyrosine phosphatase 1B, leukostasis

## Abstract

Inflammation is central to pathogenic processes in diabetes mellitus and the metabolic syndrome and particularly implicates innate immunity in the development of complications. Inflammation is a primary event in Type 1 diabetes where infectious (viral) and/or autoimmune processes initiate disease; in contrast, chronic inflammation is typical in Type 2 diabetes and is considered a sequel to increasing insulin resistance and disturbed glucose metabolism. Diabetic retinopathy (DR) is perceived as a vascular and neurodegenerative disease which occurs after some years of poorly controlled diabetes. However, many of the clinical features of DR are late events and reflect the nature of the retinal architecture and its cellular composition. Retinal microvascular disease is, in fact, an early event pathogenetically, induced by low grade, persistent leukocyte activation which causes repeated episodes of capillary occlusion and, progressive, attritional retinal ischemia. The later, overt clinical signs of DR are a consequence of the retinal ischemia. Metabolic dysregulation involving both lipid and glucose metabolism may lead to leukocyte activation. On a molecular level, we have shown that macrophage-restricted protein tyrosine phosphatase 1B (PTP1B) is a key regulator of inflammation in the metabolic syndrome involving insulin resistance and it is possible that PTP1B dysregulation may underlie retinal microvascular disease. We have also shown that adherent CCR5^+^CD11b^+^ monocyte macrophages appear to be selectively involved in retinal microvascular occlusion. In this review, we discuss the relationship between early leukocyte activation and the later features of DR, common pathogenetic processes between diabetic microvascular disease and other vascular retinopathies, the mechanisms whereby leukocyte activation is induced in hyperglycemia and dyslipidemia, the signaling mechanisms involved in diabetic microvascular disease, and possible interventions which may prevent these retinopathies. We also address a possible role for adaptive immunity in DR. Although significant improvements in treatment of DR have been made with intravitreal anti-VEGF therapy, a sizeable proportion of patients, particularly with sight-threatening macular edema, fail to respond. Alternative therapies targeting inflammatory processes may offer an advantage.

## Introduction

The advancing epidemic of diabetes mellitus, intertwined with a coincident epidemic of obesity, is one of the major public health crises facing developed and developing nations alike (1, 2). Of the two main major forms of diabetes, Type 2 far outweighs Type 1 diabetes (T1D) in prevalence and presents modern health care systems with the greater challenge (3–5). T1D is considered an autoimmune disease (6–8), possibly induced by a viral infection (9, 10) with an acute onset involving insulitis and islet cell infiltration but persisting as a chronic disease (11, 12), while Type 2 diabetes (T2D) is a chronic disease, brought on by metabolic dysregulation and insulin resistance (13, 14). Although etiologically different, both conditions are characterized by hyperglycemia and dyslipidemia which are considered major risk factors for development of the shared macrovascular and microvascular complications as well as neurological dysfunction (15); moreover, the two conditions can co-exist (4). The pathobiology of both T1D and T2D and their macrovascular complications is grounded in inflammation but there is greater debate concerning microvascular disease (16, 17). For instance, diabetic peripheral neuropathy, nephropathy, and retinopathy are microvascular complications in which small vessel endothelial cells seem to be the focus of the attack (18, 19). In contrast, macrovascular atheromatous disease is considered to be primarily a dyslipidemic/inflammatory process (20). This review examines the relationship between dysregulated metabolism and inflammation in the development of diabetic retinopathy.

## What Triggers the Development of Complications in Diabetes?

The pathogenesis of the complications of diabetes is frequently considered in terms of the cellular metabolic and signaling pathways which lead to organ dysfunction. However, signaling pathways vary between tissues and cells and so their disruption is equally tissue or cell specific. Even the vessels, particularly microvessels, are tissue specific and respond differently at each site. Bone marrow cells are also subject to the effects of the altered metabolic state of diabetes but by being distributed to all tissues, their altered functional state becomes a common tissue denominator. Thus, it has become clear that metabolically altered bone marrow derived cells contribute to organ dysfunction in diabetes (21–24). The question is do bone marrow derived cells initiate organ dysfunction and if so, do they have a direct effect on organ function or do they do so through causing vasculopathy? It is therefore important to consider altered metabolism in a cell-specific context. It follows that a second important consideration is deciding when, in the course of developing diabetes, does the altered metabolic state affect each cell type and tissue equally: specifically, once beta cell loss or dysfunction is established and hyperglycemia cannot be controlled, how well can tissues adapt to the new conditions before becoming dysfunctional or permanently damaged?

The difficulty in interpreting the pathological change lies in the lack of information concerning the initial events in diabetes and in its complications. For this reason, the United States National Institutes of Health (NIH) has initiated a prospective Human Pancreatic Analysis Programme to track the relationship between immune cell abnormality and beta cell damage (8). Meantime, some of the most informative data derive from recent collaborative studies revealing correlations between proinsulin expression and assigned insulitis lymphocyte composition in endotypes of T1D (25). Hyperglycemia is a marker for the onset of diabetes but even the threshold for this is quite wide. Normal blood glucose measurements are considered to be below 5.5 mM/L (fasting), while levels between 5.5 and 7.0 mM/L are described as pre-diabetes and a fasting blood glucose >7.0 mM/L on more than one occasion sets the diagnostic criteria for diabetes mellitus (26, 27). If endothelial dysfunction is a direct result of the systemic metabolic changes and exposure to toxic levels of glucose, do these blood levels of glucose predict changes in endothelial cell health? In culture, DNA synthesis declines markedly in retinal endothelial cells in medium containing >2.0 mM glucose (28) and is dependent on glucose uptake *via* GLUT 1 and 3. This threshold of 2.0 mM glucose is much lower than acceptable blood glucose levels, suggesting that there are other factors which maintain endothelial cell health in normoglycemia *in vivo*, i.e., up to 5.5mM/L. Indeed, there are damage limitation factors in the circulation which protect the endothelium, such as acute phase reactants (29–31) and circulating antiproteases (32, 33), while the number and activation status of the circulating cells in the hyperglycemic milieu must also have an effect (24, 34).

## When Do the Complications of Diabetes Become Irreversible?

In consideration of the onset of diabetes and when complications are likely to develop, it is probably more appropriate to envisage a progressively developing situation which is potentially reversible or repairable (35), but is one in which, gradually, insulin/leptin/glucagon-based control of central glucose and lipid homeostasis fails (36). Indeed, clinical microvascular disease in diabetes is not inevitable (37). Tissues and vessels exposed to pendulum swings of increasing metabolic stress can often adapt and survive, but in many cases, a point is reached where overt irreversible damage occurs (38). In the case of T1D, acute onset damage with loss of islet cell function takes place against a background of infectious/autoimmune inflammation in which an active immune response proceeds from the outset, and so an approximate time-of-onset can be determined. In T2D where obesity and progressive adipose tissue macrophage (ATM) activation provide the backdrop to increasing insulin resistance and low-level chronic inflammation, it is much more difficult to pinpoint either the onset of diabetes or when tissue damage occurs. In the case of diabetic retinopathy (DR), clinical signs manifest some time (often years) after the onset of diabetes; however, in T2D DR paradoxically may be the presenting sign, with the implication that an underlying diabetic state has been present but clinically “silent” for years. In fact, it is estimated that for every known case of T2D there is another undiagnosed (39, 40). The prolonged time for development of complications has been incorporated into a notion of “metabolic memory” but recent analysis has poured cold water on this idea and ascribes the risk of complications to a cumulative exposure to high glucose levels (41).

These findings emphasize the difficulty of elucidating the initial pathological events of microvascular disease in diabetes. If it is not possible to determine when the disease begins, it is even more difficult to decide what constitutes first evidence of the complications and, in a sense, this is dependent on what signs are sought and the methods used to detect them. This applies especially so to DR in which most of the classically recognized clinical and pathological signs are late and even secondary events. For this reason, experimental models, in which the onset of hyperglycemia and dysregulated lipid metabolism can be more definitively timed, at least allow determination of the earliest cellular changes and deviations from homeostasis. In addition, if it is accepted that dysregulated metabolism is the cause of the microvascular complications of diabetes, it follows that the onset of the damaging insult coincides with the onset of the metabolic change, i.e., the onset of diabetes.

## Is Inflammation in Diabetes a Direct or an Indirect Cause of Complications?

**Figure 1:**
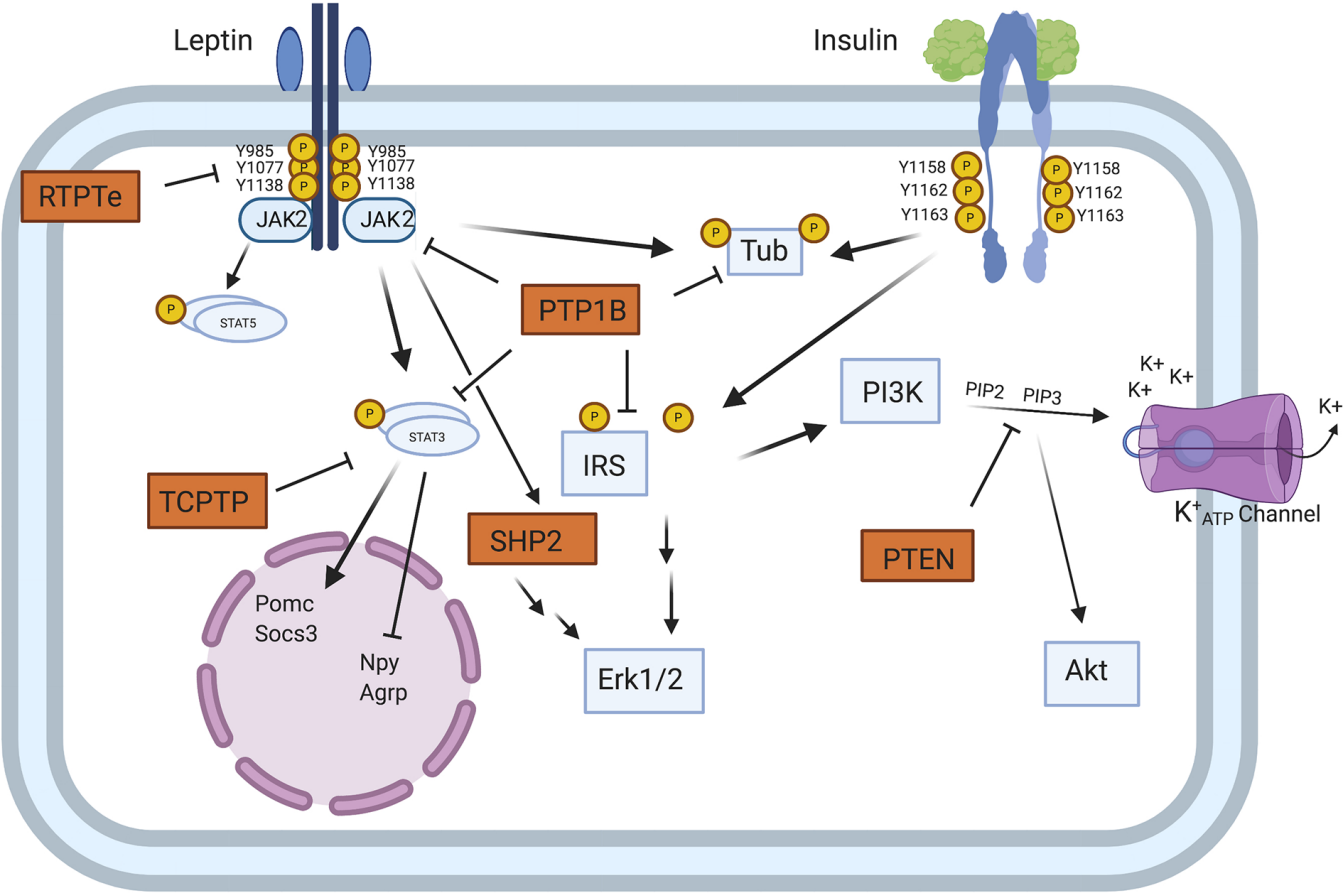
Model of PTP regulation of central leptin and insulin signaling. When circulating leptin binds to its receptor LepRb, the associated tyrosine kinase JAK2 autophosphorylates and phosphorylates specific tyrosine residues along the intracellular tail of the LepRb. Phosphorylation of Y985 allows for recruitment of the PTP SHP2 which mediates downstream ERK1/2 signaling, while phosphorylation of Y1138 allows for activation of STAT3 which regulates transcription of key neuropeptides involved in energy homeostasis. Unlike leptin signaling, insulin binding to its receptor results in receptor autophosphorylation at tyrosine residues 1158, 1162, and 1163. This allows for recruitment of the effector IRS, which upon phosphorylation can recruit adaptor molecules and mediate downstream PI3K and ERK1/2 signaling. In contrast to SHP2 which positively regulates leptin signaling, several PTPs can negatively regulate central leptin and insulin signaling. PTP1B inhibits leptin and insulin signaling by dephosphorylating JAK2 and the IR, respectively. Additionally, PTP1B has been implicated in dephosphorylating the downstream leptin/insulin signaling protein Tub. Like PTP1B, RPTPe has been shown to inhibit leptin signaling at the level of JAK2, while TCPTP negatively regulates leptin signaling *via* dephosphorylation of STAT3. PTEN antagonizes neuronal insulin-induced PI3K signaling *via* dephosphorylation of the phospholipid PIP3 into PIP2, resulting in decreased K+ ATP channel conductance [from Tsou and Bence (42)]. Figure created with Biorender.com.

The next question is whether metabolic dysregulation in diabetes is the direct cause of the complications (e.g., *via* toxic metabolites) or whether they arise from secondary effects on another system (e.g., immune/inflammatory homeostasis). The liver and adipose tissue are the engines of metabolism responsive to signals from many sources. Control of blood glucose, lipid metabolism, and body weight through insulin and leptin signaling are both peripherally and centrally regulated (42). Signaling *via* the insulin receptor/IRS pathway and the leptin/leptin receptor (LepRb) involves downstream JAK-2/STAT3/5, PI3 Kinase/Akt, and the MAP kinase pathways in hepatocytes and adipocytes with phosphorylation of the insulin receptor (InsR) and the leptin receptor (LepRb) at several sites on the cytoplasmic tails of the molecules (**Figure 1**). Recent studies have highlighted the role of microRNA’s which control the relevant signaling networks in adipose tissue, but only a few are altered in obese individuals (43). This offers an opportunity to select these specific microRNA’s as targets for therapy and/or as biomarkers.

**Figure 2:**
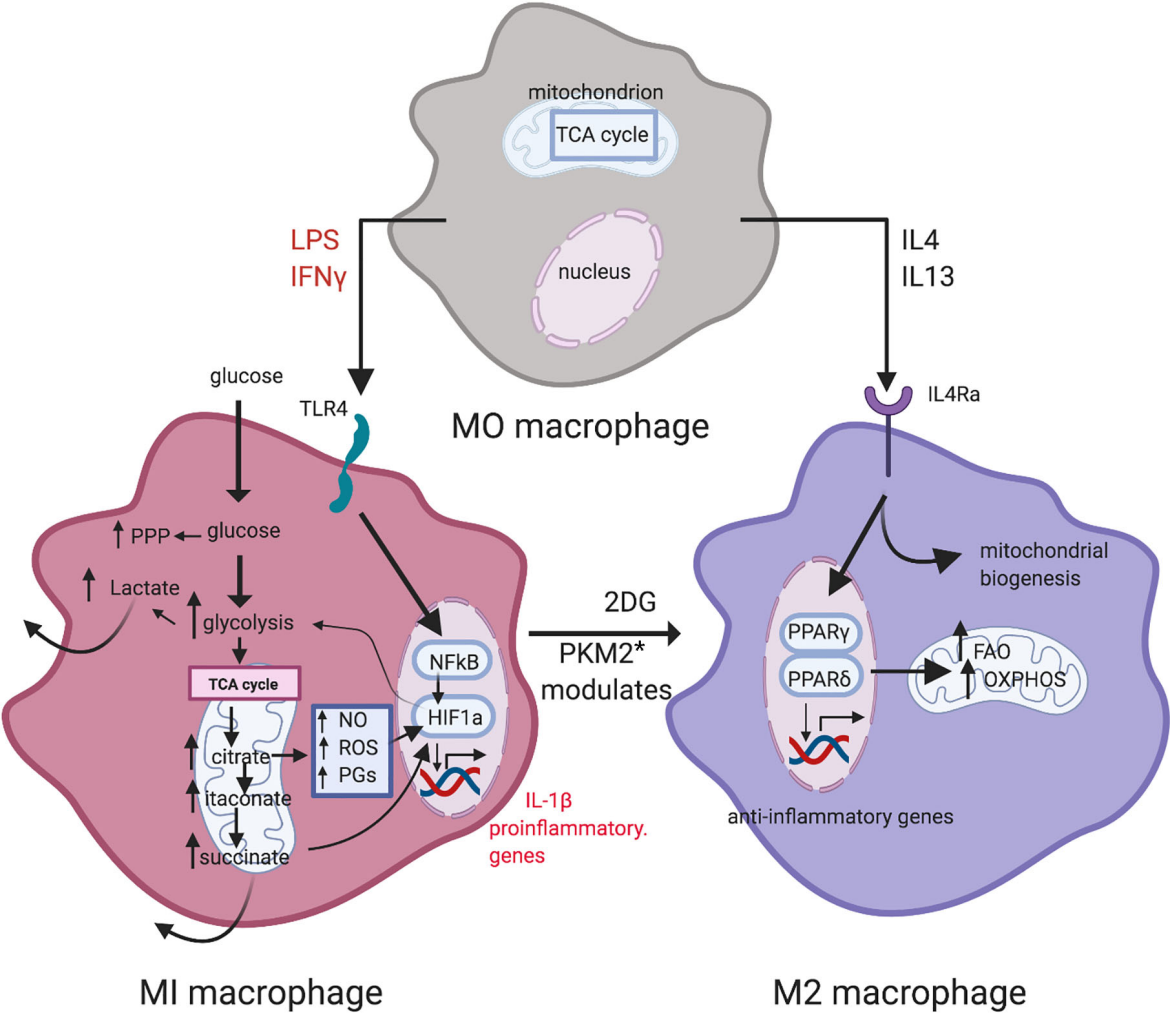
Metabolic reprogramming in macrophage polarization. LPS and IFN-γ induce M1 macrophages. Metabolism in M1 macrophages is characterized by increased glycolysis and PPP activity, and a broken TCA cycle that leads to metabolite accumulation. M2 macrophages display a more oxidative metabolic profile, with a high reliance on the TCA cycle, utilizing OXPHOS and exhibiting high levels of FAO. Inhibition of glycolysis by 2-DG leads to an oxidative M2 phenotype [from Corcoran and O’Neill (48)]. Figure created with Biorender.com. * PKM2: pyruvate kinase M2.

Regulated metabolism not only dictates the health of parenchymal tissues but underpins immune cell homeostasis (44, 45). Granulocytes have few mitochondria and do not consume oxygen to any great extent; instead, they generate ATP for energy by aerobic glycolysis (lactate production) even in states of normoxia (the Warburg effect) (46). Macrophages, dendritic cells and T cells in quiescence generate ATP for energy requirements by oxidative phosphorylation (OXPHOS) but switch to aerobic glycolysis when activated (47). In macrophages, this switch in metabolism is reflected in proinflammatory (so-called M1) vs. alternatively-activated (so-called M2) macrophages (48) (**Figure 2**). In both T1D and T2D, dysregulated glucose and lipid metabolism drive the chronic inflammatory state which manifests with increased levels of inflammatory biomarkers and acute phase reactants (29, 49, 50). Some such as C-reactive protein (CRP) and triglyceride-rich lipoproteins exert a direct pro-inflammatory effect by activating blood monocytes (20, 51).

## Insulin Resistance, Inflammation, and Diabetes

### (a) A Role for Adipose Tissue Macrophages

**Figure 3:**
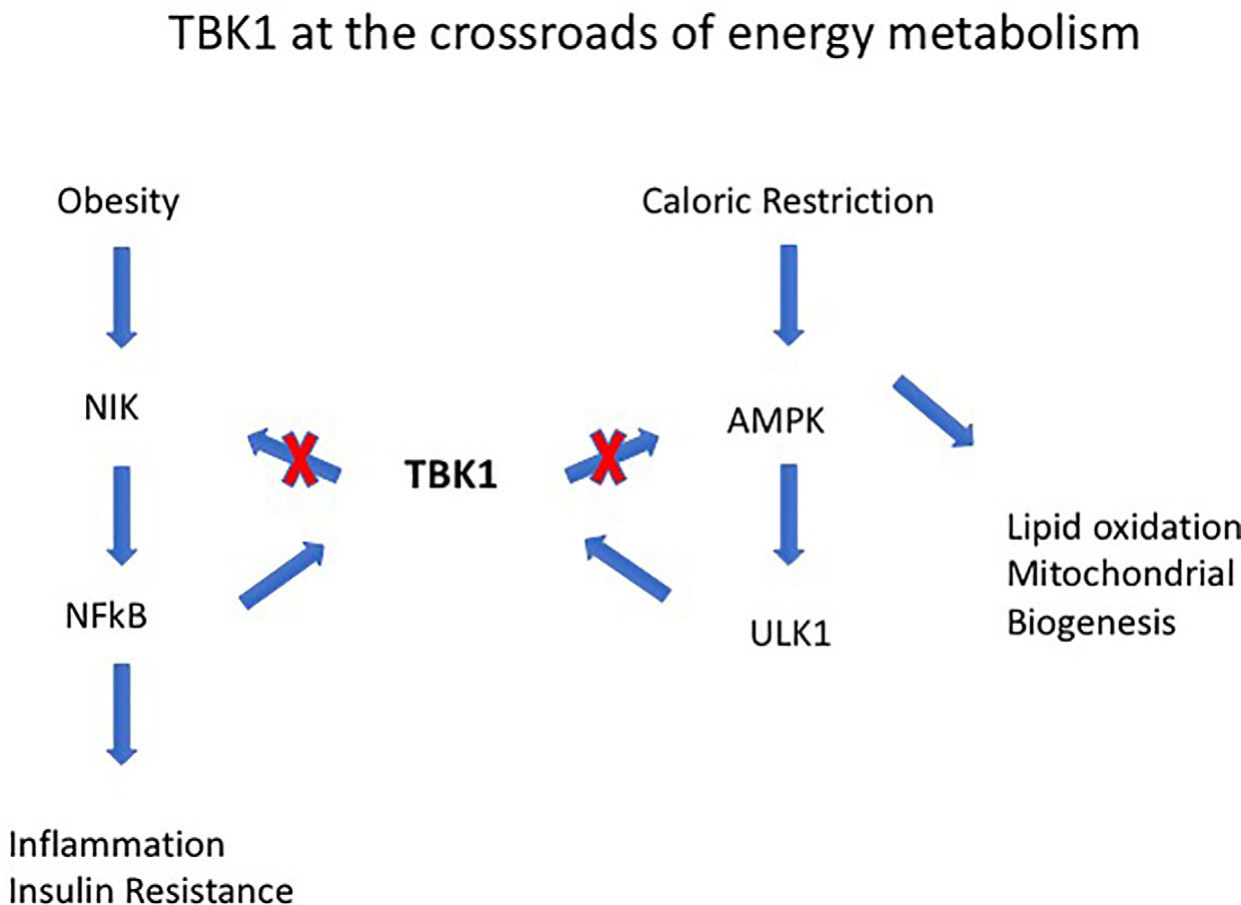
Tank-binding protein kinase 1 (TBK1) operates at the intersection of energy expenditure and inflammation. For instance, TBK1 deficiency attenuates HFD-induced obesity but exaggerates inflammation through its effects on NF-kB–inducing kinase (NIK). TBK1 represses energy expenditure by phosphorylating and inhibiting AMPK and to the serine-threonine kinase Unc-51-Like Autophagy Activating Kinase 1 (ULK1). The combined effects of TBK1 are to attenuate NF-kB activation and mediate the anti-inflammatory effect of AMPK [from Zhao et al. (58)].

Obesity-induced IR is a consequence of low-grade chronic inflammation in adipose tissue (AT) (52, 53). AT contains an extensive store of resident macrophages and dendritic cells (ATM, ATDC) whose function is to maintain tissue homeostasis. However, with increasing lipid deposition and obesity, ATM increase in number and activation status, and their cytokine secretion contributes to the low-level chronic inflammatory status associated with the metabolic syndrome (MS) and diabetes in particular (54). Activation of ATMs is mediated *via* cGAS-cGAMP-STING which, signaling through TANK binding kinase 1 (TBK1), leads to phosphorylation and nuclear translocation of the transcription factor interferon regulatory factor 3 (IRF3). This, together with nuclear factor-kappa B (NF-κB) activation and nuclear translocation, leads to production of several inflammatory cytokines including Type 1 Interferons (IFN) (55–57). TBK1 activation status in particular seems to be an important determinant of homeostasis vs. inflammation *via* ATM (58). However, the role of TBK1 may be double-edged since it promotes phosphorylation and inactivation of NFκB-inducing kinase (NIK) (58) (**Figure 3**). In contrast to ATM, the role of ATDC in obesity-induced IR and the MS is less clear. While they seem to be important in regulating AT CD4 and CD8 T cell homeostasis, disabling antigen presenting capacity in Igtx^Cre^MHC Class II^−/−^ mice had little effect on overall inflammation levels (59, 60). However, since all types of T cells, including T regulatory cells (Tregs), would fail to be activated in these experiments, the precise role of ATDC remains to be resolved.

### (b) Insulin Resistance Develops in Proportion to Adipose Tissue Macrophages

How does IR develop in AT? As adipocytes hypertrophy and accumulate increasing amounts of lipid, they become activated and secrete pro-inflammatory cytokines and chemokines, and AT, particularly visceral AT, becomes the seat of inflammation. Pro-inflammatory bone marrow derived myeloid cells, as well as T cells (cytotoxic CD8^+^, Th1 and Th17 proinflammatory cells) and NK cells, are recruited to AT while there is a concomitant reduction in the normal complement of Tregs [reviewed in (61)]. A particular subset of AT fibroblasts, the interleukin (IL)-33^+^ putative mesenchymal stem cell (mSC), increases inversely in proportion to the reduction in AT Tregs contributing to the overall increase in AT inflammation in diabetes. Potentially Tregs have the ability to reverse the deleterious effects of obesity (62) and in normal mice, over time, they become the predominant T cells in AT. Thus, an apparent protective effect of AT Tregs is lost in diabetic mice (63). At the same time, pro-inflammatory macrophages alternate with arginase^+^ alternatively activated but pro-fibrotic macrophages which, through induction by AT derived mSCs, further alter the stromal architecture of AT (64). TNFα secretion by ATMs particularly influences the development of the MS as shown by the failure of TNFα^−/−^ mice to develop diabetes and insulin resistance (IR) (65). However, arginase^+^ alternatively activated ATMs may also have a protective role through secretion of IL-10 (66–68). Precisely how adipocytes modify ATM behavior and function is unclear but recent studies suggest microRNA’s and long non-coding RNA’s (lncRNAs) may play a role. lncRNA’s have been shown to have extensive regulatory functions, mostly involving translation and transcription of proteins such as metastasis associated lung adenocarcinoma transcript 1 (Malati), Nuclear Enriched Abundant Transcript 1 (Neat) and lncDC which regulates dendritic cell differentiation *via* mir155 (69); for instance, lncDC binds to STAT3 to prevent its de-phosphorylation by SHP-1 [reviewed in (70)]. These short and long RNA moieties can be secreted from adipocytes as exosomes into the extracellular micorenvironment where they exert local control of cell behavior [reviewed in (71)]. This affects all tissues and can modify disease processes. For instance, during aging, certain lncRNAs are generated in white AT which are linked to inflammatory pathways implicating AT and obesity with age-related degenerative changes (72). In another example, pyroptosis, a process involving activation of the inflammasome, is controlled by the lncRNA NEAT1 in diabetic nephropathy through the action of the mir34c/NLRP3 axis (73). Most recently, a novel lncRNA, macrophage inflammation-suppressing transcript (74) has been described which is lost in obese mice and is reduced in ATM from obese patients (75). In sum, visceral AT and its complement of inflammatory cells, especially ATMs, is a highly dynamic cellular structure under a wide variety of intrinsic and extrinsic epigenetic controls. These become dysregulated in obesity and AT becomes a driver of systemic inflammation which is the phenotypic signature of the MS (76) and is a central feature of diabetes (77).

**Figure 4:**
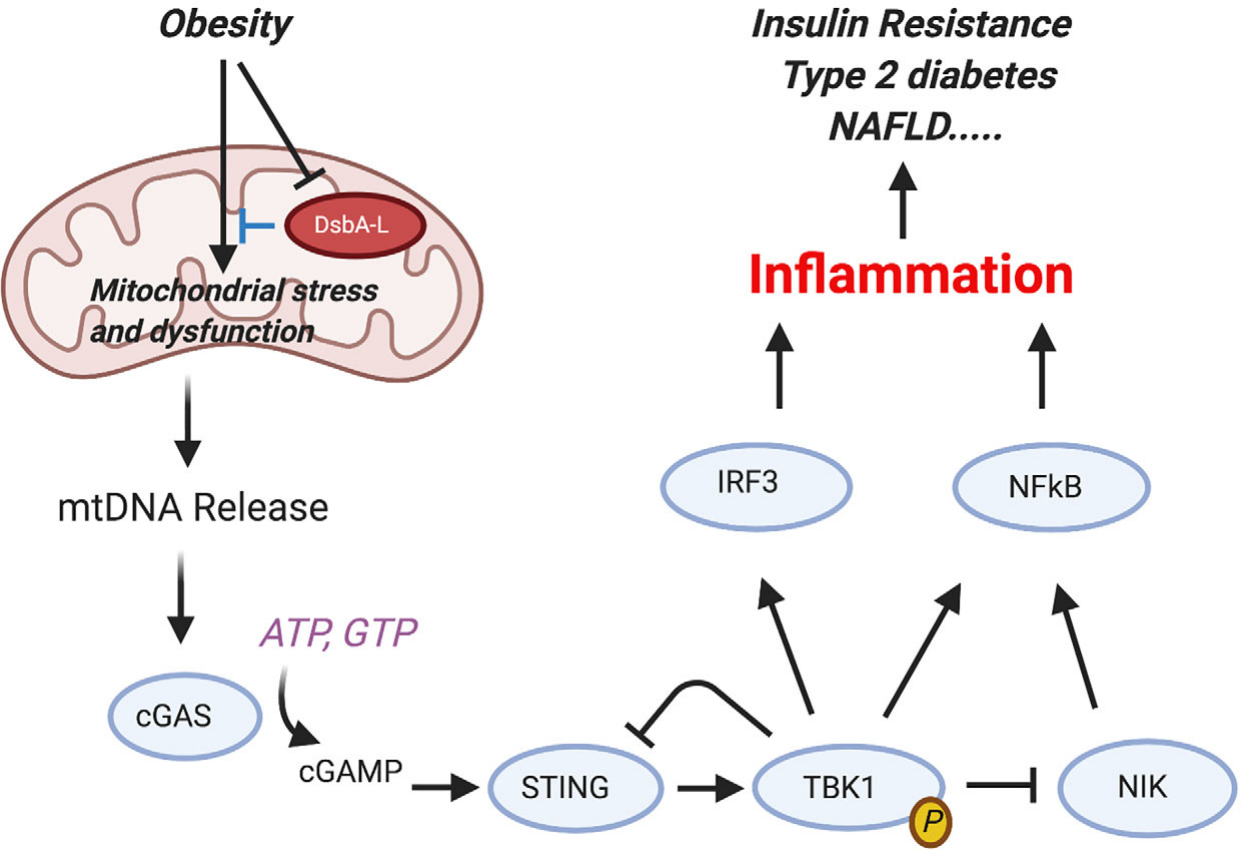
Activation of the cGAS-cGAMP-STING pathway mediates obesity-induced inflammation and metabolic disorders. Obesity reduces the expression levels of disulfide bond A oxidoreductase like protein (DsbA-L) in adipose tissue, leading to mitochondrial stress and subsequent mtDNA release into the cytosol. Aberrant localization of mtDNA in the cytosol activates the cGAS-cGAMPSTING pathway, leading to enhanced inflammatory gene expression and insulin resistance. Phosphorylated and activated TBK1 exerts a feedback inhibitory role by promoting STING ubiquitination and degradation or stimulating phosphorylation-dependent degradation of NF-kB–inducing kinase (NIK), thus attenuating cGAS-cGAMP STING–mediated inflammatory response [from Bai and Liu (80)]. Figure created with Biorender.com.

Importantly, these AT changes and the development of the MS often precede overt diabetes. Experimentally the MS can be modelled by dietary manipulation, as in the high-fat diet (HFD) mouse model (78). Such mice have increased serum adipokines, elevated triglycerides and altered levels of HDL and LDL cholesterol in blood, IR, and transient hyperglycemia, and go on to develop signs of atherosclerosis (79). In addition, they have a pro-inflammatory systemic signature. Metabolic activity in macrophages and DC is regulated through activity of kinases and phosphatases but differentially from parenchymal (e.g., liver) cells. Each cell type in fact plays a specific role. For instance, TBK1 by phosphorylating mTORC-1 exerts a negative feedback effect on STING (80) and thus exerts a dual role on macrophage-induced inflammation (**Figure 4**). In contrast, we have shown that deletion of macrophage specific protein tyrosine phosphatase-1B (PTP1B) in LysM-PTP1B mice markedly reduces HFD-associated inflammation and also protects mice against LPS-induced endotoxemia (81). These effects were associated with increased levels of phosphorylated (p)-STAT3 and increased production of IL-10. Thus, regulation of metabolism (body weight) and the associated inflammation (endotoxemia, leukocyte activation) appear to be under control of resident ATMs rather than adipocytes and hepatocytes *per se*.

### (c) Are Insulin Resistance and Chronic Inflammation the Host’s Defense Against Overnutrition?

What is the stimulus for bone marrow derived macrophages (BMDMs) and ATMs to shift to a pro-inflammatory mode? As Donath et al. put it “…… three key features of T2D—namely, insulin resistance, decreased insulin secretion and glycosuria—are primarily mechanisms that protect against overnutrition by preventing the accumulation and overloading of tissues with cell nutrients” (82). IL-1 stimulation of insulin secretion by pancreatic beta cells *via* diacyl glycerol protein kinase C signaling underpins this concept (83). Islet beta cells have high levels of IL-1r and in response to increased glucose undergo inflammasome activation and secrete IL-1, thereby attracting further islet infiltration by macrophages which sets a positive feedback loop for IL-1 secretion [see ref. (82)]. Increased glucose metabolism and hexokinase activity alone in macrophages are sufficient to act as pathogen/damage associated molecular patterns (PAMP/DAMP) with production of pro-inflammatory cytokines, particularly IL-1 (84) and this can even be induced transiently by postprandial hyperglycemia (85). This sets the wheels rolling for progressive beta cell apoptosis, insulin loss/resistance, and persistent low level chronic local and systemic inflammation which begin when ambient glucose levels are above a threshold that cells can manage. However, loss of pancreatic insulin in T1D and IR in T2D with their consequent effects on glucose and lipid metabolism eventually leads to ATM activation and increase in numbers. This is caused by adipocyte increase in size, nutrient overload and apoptosis which, as indicated above, further attracts monocyte/macrophage infiltration (86).

ATM precursors/monocytes respond to pro-inflammatory signals and arrive at their target tissue in an activated state (23). Thus, circulating monocytes in obese and/or diabetic individuals and in HFD fed/diabetic experimental animals express markers of activation such as increased levels of L-selectin, ROS and iNOS (87–91). Monocyte/myeloid cell activation occurs in the early stages of diabetes and HFD-induced obesity, and inflammatory biomarkers are elevated before the onset of overt diabetes (92, 93). Although there may be some local replication of F4/80^+^ yolk sac-derived resident tissue macrophages (94), inflammatory macrophages such as those seeding AT, as well as endothelial progenitor cells (EPCs) which sustain vascular health (see below) (95), are mostly bone marrow-derived (24, 96).

This process, i.e., recurrent cycles of immune cell activation and inflammation, not only begins at the onset of the MS and obesity/HFD disease, but actually represents the onset of these disorders when the threshold of homeostasis is breached. The logical conclusion is that this pro-inflammatory backdrop may be the driver for the overt diabetic state and its complications (82). The recent studies showing the benefit of anti–IL-1 antibodies in preventing macrovascular complications of diabetes support this re-think (97, 98). As the disease progresses, other factors contribute to and exacerbate the underlying pro-inflammatory condition. These include AT hypoxia, induced by the expansion of tissue outstripping its capillary vascularization which may in fact be a result of capillary occlusion and loss, due to intravascular leukocyte entrapment and coagulation. The resultant tissue hypoxia promotes further macrophage infiltration. In addition, alterations to the gut microbiota which is a common feature in diabetic patients are associated with increased gut barrier leakiness and the entry of pro-inflammatory bacterial products to the system (99, 100).

Glucose homeostasis is finely balanced and the mild post-prandial transient inflammatory response associated with glucose intake may in fact be beneficial and represent a form of parainflammation (101, 102). The trouble begins when this adaptive mechanism breaks down.

## Primary and Secondary Features of Diabetic Retinopathy

The above preamble is especially relevant to the pathogenesis of DR, since DR develops against a background of chronic systemic inflammation. It follows that circulating inflammatory mediators will modify retinal function. Altered glucose metabolism has primary and secondary effects on the retina. Primary effects are the result of disturbed glucose and lipid metabolism directly affecting retinal cells including neural cells, supporting cells (glia, microglia, Müller cells) and vascular cells, including endothelial cells, pericytes as well as intravascular cells. Secondary effects of diabetes on the retina are the consequences of the primary insults.

Thus early functional changes to the retina in diabetes include characteristic changes in the electroretinogram (103) as well as defects in autoregulation of vascular blood flow (104) which may be detectable before clinically visible signs of retinopathy appear. In addition, there are direct neurodegenerative changes to glial cells (105, 106) and to photoreceptors (107, 108). Recent reviews of diabetic retinopathy faithfully document the many pro-inflammatory changes which occur in the retina, involving nitric oxide (NO), cyclo-oxygenase (109), leukotrienes, vascular endothelial growth factor (VEGF) and more but do not highlight the fact that one of the central pro-inflammatory mediators is glucose at high concentration (110). Not only will an altered systemic metabolism of diabetes directly affect retinal tissues leading to sequential changes in all these other mediators, it is also the case that both the retinal circulating leukocytes as well as the retinal vascular endothelium will not be spared and will have secondary retinal damaging effects from the onset of diabetes. Indeed, the constant trafficking of hyperglycemia-activated pro-inflammatory leukocytes through a retina in contact with endothelial cells bathed in toxic levels of high blood sugar is inevitably bound to have a deleterious effect.

**Figure 5:**
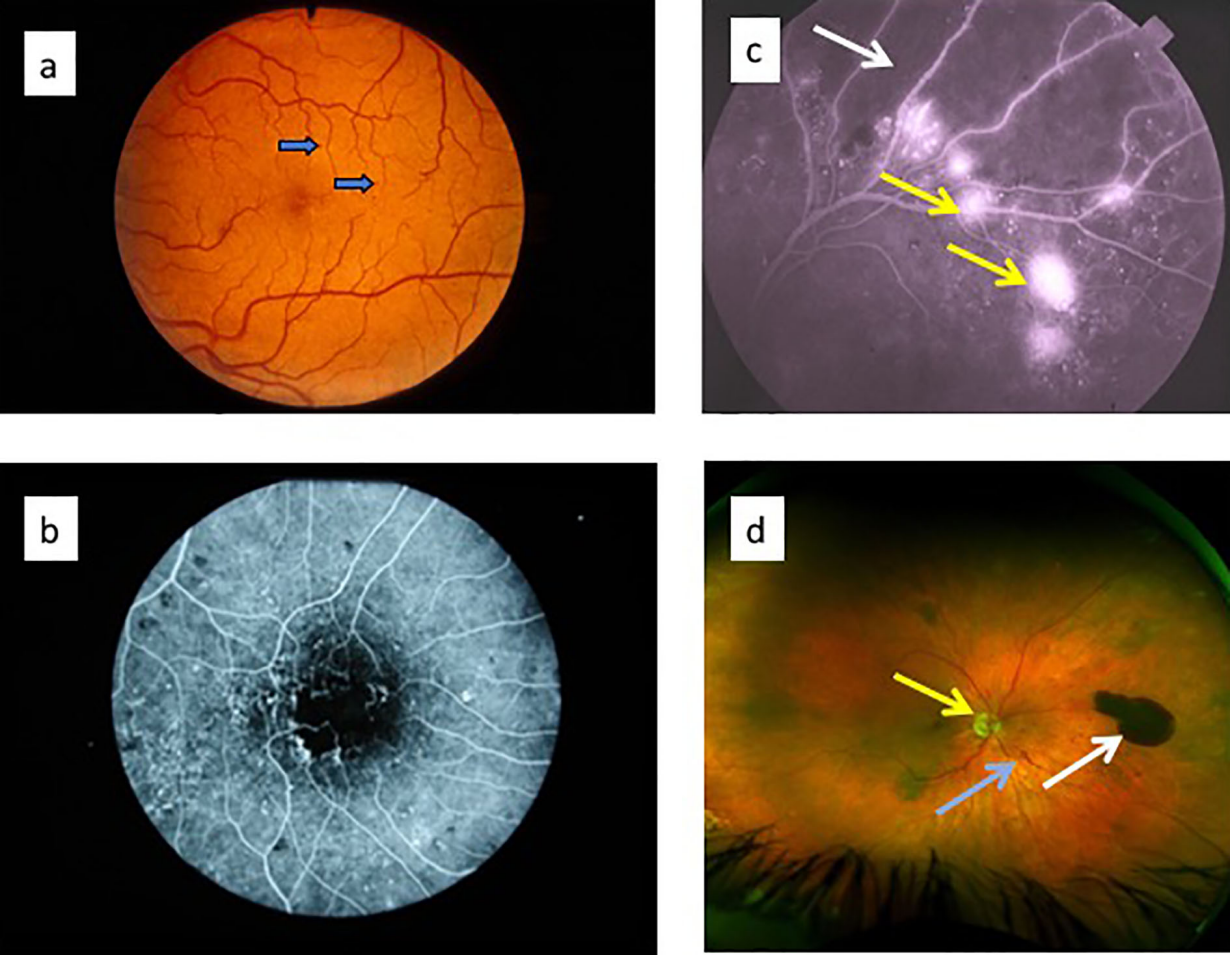
Retinal fundus images of diabetic retinopathy. **(a)** fundus photograph showing microaneurysms (arrows); **(b)** late fluorescein angiogram showing numerous microaneurysms (white dots) with small surrounding dark patches indicating focal retinal ischemia; **(c)** late fluorescein angiogram showing abnormal capillary permeability as small areas of diffuse dye leakage from microaneurysms (yellow arrows) surrounding a large dark, non-perfused patch of retinal ischemia (white arrow); **(d)** ultrawide field fundus image showing optic nerve head (yellow arrow), intraretinal microvascular abnormality (blue arrow) and pre-retinal haemorrhage (white arrow) (112).

**Figure 6:**
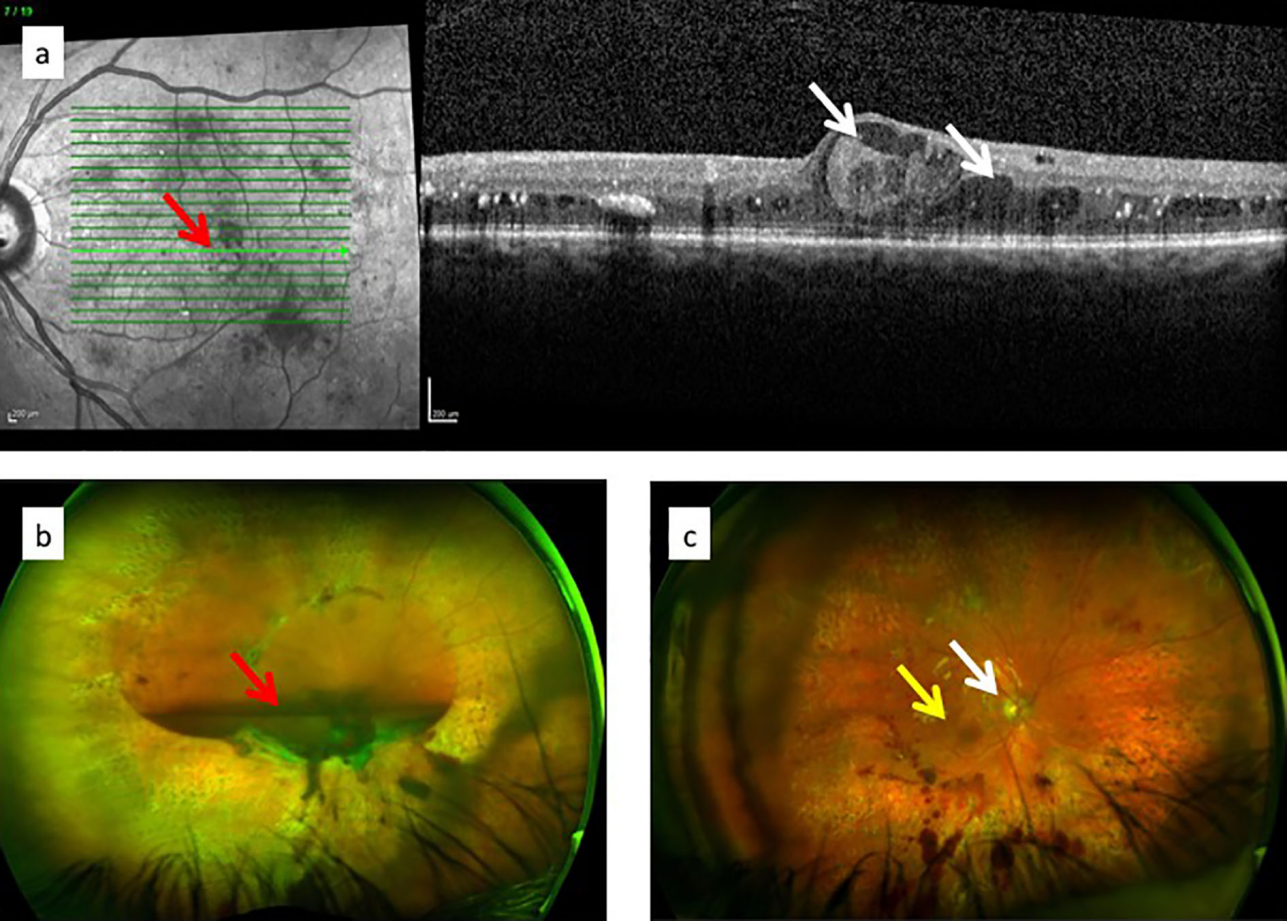
**(a)** Optical coherence tomography (OCT) images of diabetic macular oedema: fundus image to indicate plane of “optical section” (red arrow) through the retina showing macular oedema/retinal cysts (white arrows) in image on the right; **(c)** ultrawide angle, pre-surgical image of pre-retinal (subhyaloid) haemorrhage in the vitreous cavity obscuring the optic nerve and macula; **(c)** post-surgical view of same eye in **(b)**: vitreous cavity is now clear and optic nerve head (white arrow) and macula (yellow arrow) are visible.

The classical clinical signs of DR are well known (**Box 1**). Microaneurysms (m/a) are the hallmark of DR and the condition cannot be diagnosed in their absence (**Figure 5a**). However, m/a are also a feature of other retinal diseases such as hypertensive retinopathy (HR) and they also occur in non-retinal tissues. M/a are surrounded by small but increasing areas of retinal ischemia, which is clinically detectable by retinal angiography using a fluorescent dye. Thus focal micro-patches of retinal ischemia are prominent features in the early stages of microvascular retinopathy (**Figure 5b**). An associated early sign is breakdown of the blood retinal barrier evidenced clinically on angiography as fluorescein dye leakage which permeates retinal tissues and spills over into the vitreous cavity (**Figure 5c**). Progressively larger areas of retinal ischemia in time are followed by intraretinal microvascular abnormalities (IRMA) (**Figure 5d**) leading to neovascularisation/proliferative DR (PDR, **Box 1**) with crops of new vessels sprouting from the optic nerve head and the mid-peripheral retinal venules. These vessels ultimately haemorrhage into the vitreous cavity due to fibrotic/gliotic vitreoretinal adhesion and traction, and finally end with retinal detachment (**Figures 6b, c**) (**Box 1**). Severe non-proliferative DR (NPDR) is characterized by increasing retinal oedema, venous abnormalities (“beading”) (**Figure 6**) and deposits of lipid-rich exudates often concentrated around the macula/fovea. Both PDR and macular edema (**Figure 6a**) are driven by high local levels of hypoxia and release of VEGF (110, 113–115). Sight loss from macular edema is typical of T2D while intraocular haemorrhage and tractional retinal detachment are more common in T1D, although both causes of sight loss frequently occur in either type of diabetes. In terms of frequency therefore macular edema is the more common cause of visual disability related to diabetes.

Box 1 Clinical Signs of Diabetic Retinopathy (DR)*.

The signs of DR normally develop sometime (years) after the onset of diabetes. However, they do not occur in all patients (111), and some subsets of diabetes may be protected from DR. DR is uncommon in childhood diabetes but typically develops in adolescence. The clinical features progress as seen by ophthalmoscopy, fundus fluorescein angiography (FFA) optical coherence tomography (OCT) and OCT-angiography (OCT-A) are described below.

**Table:** 

Clinical finding by ophthalmoscopy*	FFA	OCT/OCT-A
microaneurysm	focal ischemia	
dot haemorrhage	fluorescein dye leakage	patchy thickening
blot haemorrhage	capillary non-perfusion	capillary non-perfusion
venous irregularity		
intraretinal microvascular abnormality (IRMA)	increased dye leakage increased non-perfusion	increased thickening increased non-perfusion
gliosis		retinal surface traction
neovascularization of optic nerve head/periphery (PDR **)	severe dye leakage	severe non-perfusion
retinal thickening/macular edema	extensive dye leakage	increased retinal thickness, intraretinal cysts
tractional retinal detachment	severe dye leakage severe non-perfusion	retinal elevation retinal traction

*Figures 5, **6** ** PDR: proliferative diabetic retinopathy.

All of the above features of DR are late events, including sight loss (which is a very late event) since they develop at variably prolonged times after the onset of hyperglycemia and dyslipidemia. M/a [so-called “background retinopathy” (95)] do not appear until years of diabetes have elapsed. Micropatches of retinal ischemia are not detected unless fluorescein angiography is performed which is rarely clinically indicated in a normally sighted individual. It is possible that with new imaging techniques such as optical coherence tomography angiography (OCT-A), research projects to identify the earliest clinical signs might uncover more subtle vascular signs but there are limited data so far (116). Interestingly OCT-A studies confirm that capillary vessel changes occur prior to the onset of clinically overt diabetic retinopathy. Historically, pathological studies consider pericyte loss to be the earliest sign in retinal capillaries, with capillary “drop-out”, acellular capillaries and microaneurysms also prominent features [reviewed in (117)]. However, post-mortem trypsin digest, retinal flat mount studies on samples with minimal signs of DR are usually performed sometime after the onset of diabetes.

Experimental models of DR do not fully replicate the features of DR in humans, particularly the later changes of PDR. In addition, only primates have a macula thus limiting studies of macular oedema. However, microaneurysms and retinal capillary non-perfusion/ischemia are features of DR in the mouse and rat (87). Several models of diabetes in rodents are available (118) but the most frequently used is the streptozotocin (STZ)-induced model. The model is criticized for the systemic pro-inflammatory effects of STZ but its chemical toxicity is relatively islet specific and by causing selective death of beta cells resembles both Type 1 and Type 2 diabetes. Its pro-inflammatory effects may be due to beta cell destruction as in T1D (see above). In addition, by modifying the induction protocol, both acute and chronic models can be established in the STZ mouse, the latter being the preferred model, and the time of onset of diabetes (hyperglycemia) can be definitively set.

Clinical signs of DR in the STZ mouse are few. Occasional m/a can be detected. Prior to the introduction of rodent fundus imaging techniques (119, 120), the earliest signs of DR such as pericyte loss and capillary drop out were not detected until several months after onset of diabetes.

## Diabetic Retinopathy Is Primarily a Vascular Disease in Which the Endothelium Is the Target of Attack

**Figure 7:**
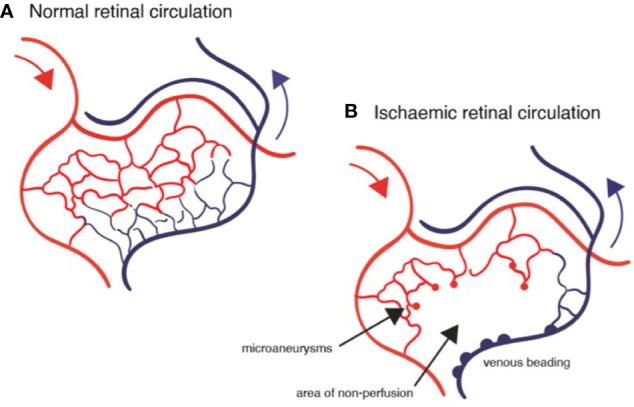
Diagrams of retinal circulation: **(A)** normal blood flow from arterioles (red) through capillary network to venules; **(B)** abnormal blood flow in diabetic retinopathy showing microaneurysms, capillary loss (drop-out), retinal ischemia and venous beading.

**Figure 8:**
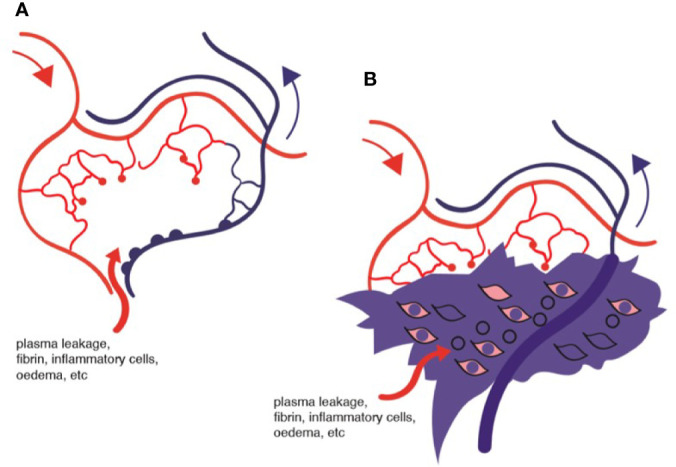
Diagram of ischemic retina: **(A)** area of ischemia and oedema; **(B)** representation of tissue fibrin deposition and macrophage infiltration surrounding non-flowing venule.

Because the clinical features of DR predominantly involve changes in the retinal vessels, DR is considered by many to be primarily a small vessel disease caused principally by endothelial cell damage due to disrupted metabolism. There is little doubt that the vascular changes modify retinal function since, as Rafii et al. say, capillary endothelial cells are the “gatekeepers of cellular metabolism” in the tissues (121). Endothelial cells also have a secondary role in providing trophic and other factors essential to tissue health which is unrelated to their role in tissue perfusion and nutrient transport functions. This “angiocrine” function is mediated by factors such as VEGF, fibroblast growth factor (FGF), thrombospondin (TSP)-1, and others and is tissue specific [reviewed in (121)]. The neurovascular unit of the CNS (122) probably fulfils this role in the retina. In diabetes, capillary endothelial cells are liable to damage with leakage of glucose-rich plasma into retinal tissues (**Figures 7** and **8**) and an increased retinal cell glucose flux with generation of reactive oxygen and nitrous oxide species (87, 123–125). Photoreceptor dysfunction (108), Müller cell/glial cell activation with gliosis (105, 126), and ganglion cell loss with overall retinal thinning are well documented (108, 127). However, these events occur after sustained initial damage to the capillaries (years in humans, months in rodent models) and are therefore secondary to microvascular disease. In addition, they do not correlate directly with vascular damage (108). The question therefore is whether the microvascular disease is primarily an endotheliopathy due to direct glycolipid toxicity or is due to a ramping up of the MS and systemic inflammation leading to secondary endothelial damage. This important question has implications for therapy.

The classic signs of pericyte loss, basement membrane thickening and acellular capillaries, described as the earliest pathological changes of DR in humans (117) also occur in the STZ mouse. As in humans, they are reported as some of the first signs but they are not early events since they occur months after the onset of hyperglycemia and diabetes. In addition, they are not unique to the retina: they are in fact manifestations of a diabetes-related vasculopathy also seen in many tissues, including the choroid (128, 129), the heart (130, 131), peripheral nerves (18), and the kidney (19). Moreover, retinal microaneurysms, acellular capillaries, and other vascular changes occur in the absence of diabetes, for instance, in aging (132) and hypertension (133). Thus, while the microvascular changes are characteristic of DR they are not specific and might be better considered caused by several mechanisms, one of which is glycolipid toxicity of diabetes. The question then is how do these various insults cause microvascular disease and what are the initial events?

For obvious reasons, the earliest pathological signs of microvascular DR in humans are difficult to record. Experimentally, both *in vivo* and *in vitro* loss of endothelial cells and pericytes due to apoptosis is a recognized consequence of exposure to high glucose levels (125, 134, 135). Endothelial cells have a defined life span and in healthy individuals there is a natural turnover, replenished from bone marrow precursors (136, 137). Endothelial cells are subject to the constant flow of blood under pressure and particularly at branching points are exposed to stress. Capillaries accommodate only a single file of flowing cells with individual leukocytes interspersed within columns of red cells (rouleaux). This has recently been visualized in the retinal capillaries *in vivo* non-invasively using adaptive optics (138) and periods of red blood flow stasis of several second duration were observed and attributed to “interactions” with leukocytes as well as turbulence at capillary branching points. The passage of leukocytes in retinal vessels also explains visual entoptic phenomena (104). Capillaries are dynamic structures and can respond to the demands of the tissue by changing their perfusion status (139). This is particularly seen in the retina where the perifoveal capillary network can be seen to undergo changes in its angiographic pattern as shown very clearly on OCT-A (139). In diabetes, these changes are more pronounced (140) and pathologies such as microaneurysms, vascular leakage and dot and blot haemorrhages come and go as vessels open and close (141). Indeed it is also possible for areas of vascular occlusion and retinal non-perfusion to become re-perfused in early diabetic retinopathy (142). Reperfusion occurrs *via* two mechanisms: recanalization and focal intraretinal neovascularization (see above, IRMA). It can be seen therefore that permanent changes such as pericyte loss and acellular capillaries are akin to findings from an archaeological dig, representing damage and decay from events long past.

There is no argument concerning the presence of vascular damage in diabetes. Endothelial cells are under stress and tend to apoptosis, an effect which could be due to the direct effects of high glucose (28) or to reactive oxygen or nitrous oxide species (125). A reduction in tissue perfusion and oxygenation, due to lack of blood flow in capillaries which have lost their endothelial lining will unquestionably lead to tissue dysfunction, which particularly applies to the retina with its high oxygen usage (139). In addition, capillary leakage, and fibrin deposition in the extracellular ischemic space will compound the pathology (**Figure 8**). The question is: what causes the capillary non-perfusion? Is it a chemical toxicity to the vascular endothelium and supporting pericytes caused by exposure to high levels of glucose and dyslipidemia of diabetes, thus leading to capillary dysfunction and inability to allow blood stream flow? Or is there a sustained intravascular obstruction to flow?

As detailed above, the clinico-pathological signs of DR can also be reproduced in other clinical diseases and experimental situations. Severe HR has many of the features of advanced DR with haemorrhages, capillary closure and nonperfusion, venular dilatation, and ischemia and, as for the early stages of DR, these changes are reversible if the blood pressure can be controlled. In addition, experimental models of HR document similar retinal changes pathologically (143). Furthermore, hyperlipidemia (HFD) without diabetes or raised blood pressure also generates a similar pathology suggesting that the metabolic changes alone are sufficient to cause the vasculopathy (79, 144). The question therefore remains whether the vessel is faulty or the vessel contents are at fault. However, this may be less important in practice than it appears since once the metabolic changes become pathogenetic, both endothelial cell and blood leukocyte physiology will be altered, probably simultaneously, thus promoting abnormal leukocyte-endothelial interactions, i.e., adhesion.

## Diabetic Retinopathy Is an Inflammatory Disease

The earliest clinical feature of DR, i.e., the m/a, highlights the dynamic nature of the disease. How m/a form is unclear but they are considered to be regions of vessel wall dilatation or outpouchings. However, an alternative view is that they are blind ends of occluded capillaries, often at branching points, representing aborted attempts at revascularisation/neovascularization (117) (**Figures 5c, d** and **6b**). As stated above, m/a are associated with micropatches of retinal ischemia (non-perfusion) which in essence equate to areas of focal capillary occlusion. M/a also, as stated above, come and go (141) indicating that reperfusion of discrete areas of retinal ischemia has taken place. Indeed a reperfusion of ischemic retina has been documented by fluorescein angiography (142). This is particularly evident in the early stages of DR but as time goes by the numbers of m/a and the areas of retinal ischemia increase. This suggests an attritional process of microvascular occlusion which is best explained by increased numbers of non-perfused capillaries each trapped at different times by a single adherent leukocyte as dictated by capillary dimensions (145). This is termed leukostasis but is more correctly described as flow stasis caused by trapped/adherent leukocytes. Cells travel in single file in capillaries and transient leukocyte arrest occurs in healthy individuals albeit only in occasional vessels and usually at branching points due to turbulent flow. Capillary non-perfusion/reperfusion also occurs in healthy retina and indeed is a process of dynamic vascular remodelling essential to retinal health (see above) (139). In DR, this process falters and intravascular leukocyte arrest becomes more frequent and prolonged, and sometimes irreversible, with capillary occlusion, vascular leakage, retinal ischemia and damage, and hypoxia, leading eventually to the later secondary changes of severe NPDR and PDR (see **Box 1**). In the early stages, remodelling and reperfusion are possible but with time the ailing vasculature cannot cope with the constant barrage of circulating, adhesive leukocytes.

It is important to consider that increased leukocyte trapping/leukostasis parallels the increase in blood glucose/dyslipidemia (146, 147) but this has been attributed by some authors to changes in the endothelium rather than the circulating leukocytes (148). As detailed above, hyperglycemia activates circulating leukocytes which are then liable to adhere to vessels walls (149, 150). Just as there is a progression from glucose homeostasis through prediabetes to overt disease, there is a coincident change in leukocyte/vessel interaction, from occasional transient leukocyte trapping to multiple sites of prolonged leukostasis with fewer successful reperfusions, until eventually networks of capillaries are permanently occluded, lose their endothelial lining and pathologically present as areas of reduced capillary density and acellular capillaries in the later stages of DR (151) (**Figures 5** and **7**).

The concept of activated leukocytes causing capillary occlusion was first proposed by Schroder et al. (152) who demonstrated trapped granulocytes and monocytes within retinal capillaries. Further studies found that increased levels of circulating soluble adhesion molecules (ICAM-1, VCAM-1, P-selectin) not only occurred in diabetes but correlated with the severity of DR (91). Importantly, increased levels of adhesion molecules (ICAM, VCAM and PECAM) were often present contemporaneously with the onset of diabetes, were closely related to the level of HbA1c and were considered to be measures of endothelial cell damage (153). Soluble adhesion molecules are shed from endothelial cell surfaces usually after contact with flowing cells. In the circulation, increasing levels are more likely a measure of the increasing number of “hits” in terms leukocyte-endothelial cell adhesive interactions, which does not occur in health. In diabetes, obesity, and the MS, circulating leukocytes show signs of activation related to chronic low levels of systemic inflammation, driven either by beta cell loss or IR (see *Is Inflammation in Diabetes a Direct or an Indirect Cause of Complications* above). This manifests as increased levels of reactive oxygen species (ROS) (123, 154) and iNOS (87) as well as increased surface levels of the integrin CD11b (155) and other molecules (see below). Increased circulating ROS directly damage the vascular endothelial surface, specifically its hyaluronan - rich protective glycocalyx, which depolymerises and thins, eventually exposing the plasma cell membrane to direct contact with activated leukocytes (156, 157).

Increased leukocyte-endothelial cell adhesion was inferred from experiments showing that leukostasis was reduced both by the blockade of adhesion molecules using monoclonal antibodies as well as in ICAM^−/−^ and VCAM^−/−^ mice [reviewed in (106)]. Such mice also had reduced late vascular changes of DR including reduced pericyte loss and acellular capillaries compared to untreated diabetic mice. However, increased expression of adhesion molecules on the endothelial cell surface is not necessarily a measure of inflammation and could simply be an expression of disordered metabolic state of the cells due to diabetes. More compelling evidence of a primary role for inflammation in DR rests on the activated state of circulating leukocytes. Activation of innate immune cells is reflected in increased levels of co-stimulatory molecules such as CD40, CD80, and CD86 as well as MHC Class II. In myeloid cells generally, increases in CD11a, b and c also indicate heightened activation status. Both hyperglycemia and hyperinsulinemia can increase CD40 levels in monocytes and platelets (158) while inhibition of CD40 expression prevents both leukostasis and ICAM-1 expression in endothelial cells, as well as late capillary loss/degeneration and inflammation (159, 160). Increased levels not only of CD40/CD40L but Toll-like receptors, ER stress, CCR5 and Fc-*γ* receptors (CD32 and CD64) are characteristic of the MS (161), and specifically, the CD11b^+^CCR5^hi^ monocyte is preferentially involved in early onset STZ diabetes-induced leukostasis (145). The pro-inflammatory profile of monocytes in diabetes, including increased expression of CD80, has long been recognized (162). However, it is important to realize that chronic inflammation in diabetes is dysregulated; indeed, the performance of inflammatory cells in responding to acute disease/injury is impaired in patients with diabetes compared to healthy individuals, perhaps related to skewed myelopoiesis (24, 163).

Overall, these correlations although compelling are not definitive proof that activated leukocytes, and by inference a background of inflammation, are the cause of the retinal non-perfusion and ischemia in diabetes and MS. Ischemic retina is widely accepted as a source of VEGF, either from retinal cells such as Müller cells (164) and ganglion cells (165) or from the activated leukocytes themselves (166). It is also possible that the activated leukocytes induce Müller cell production of VEGF (167). The beneficial effects of blocking VEGF in DR clearly identify this mechanism. VEGF is known to be a major player in inflammation and so inflammation as the root cause of pathology, including the pathology of DR remains a strong concept. However, the situation is complex since VEGF exacerbates leukostasis and blocking the VEGF receptor permits retinal reperfusion (168). Only by preventing leukocyte-driven inflammation can the question be resolved.

A deeper look at leukostasis/leukocyte arrest generally is important since it occurs infrequently in healthy vessels, small and large. All vessels are subject to the stress of blood flow and in capillaries the constant intimate contact with trafficking cells. Indeed the endothelial hyaluronan-rich glycocalyx appears designed to protect the endothelium from flow-cell damage (156, 157). Endothelial cells therefore require a mechanism for repair/turnover which is provided by EPCs (169). Both haemopoietic stem cells (170) and EPC arise from a common source embryologically [termed the haemothelium (171)] and in the bone marrow occupy separate niches (95). HSC-derived stromal macrophages guide vascular EPCs to differentiate and migrate/mobilize into the circulation. Here, these rare cells are attracted to sites of endothelial injury and settle contiguously into the existing monolayer.

Evidence for this repair process has been suggested most recently, in a study of aging and endothelial cell turnover in retinal vessels. As the endothelial cell becomes senescent, neutrophil leukocytes adhere to the dysfunctional cell and engage in its removal (172). Apparently, the aging endothelium sends out a “secretome” to attract neutrophils which, as activated cells, release neutrophil extracellular traps (NETS) to surround and clear the diseased cell. This allows remodelling of the vessels which is achieved by replacement of the defective endothelial cell by circulating/rolling EPCs as suggested above. Diabetes is considered a type of accelerated aging (173, 174) and this mechanism of endothelial repair might be overactive in diabetic retinal vascular disease. Indeed, it is possible that defects in this repair mechanism may underlie microvascular occlusive retinal vascular disease as suggested above. If this is the case, then the primary abnormality would be attributable to the endothelium in diabetic vascular disease, but would depend on normal, effective leukocyte activation and NETosis to prevent disease.

However, in diabetes, the source of myeloid leukocytes (i.e., the bone marrow) is a target tissue, not only causing hyperglycemic activation of leukocyte progenitors but impairing EPC mobility and preventing adequate repair of damaged vascular endothelium. In addition, the balance between bone marrow EPC’s and HSC’s is disturbed (175). It is possible that the increased leukostasis observed in DR represents a failure of EPC-mediated repair by activated myeloid progenitors to fill the gap. The result is however, the opposite, in that the activated, but dysregulated, leukocyte causes further damage to the endothelium with progression to an acellular capillary.

In this scenario, a damaged, prematurely aged endothelium might be seen as the primary abnormality in the microvascular complications, particularly retinopathy, of metabolic disease including diabetes. However, this may be a somewhat pointless discussion since both players, i.e., the activated leukocyte and the damaged endothelium, are both metabolically disturbed from the onset of disease.

## Leukocyte Activation in Diabetes—What is the Evidence?

**Figure 9:**
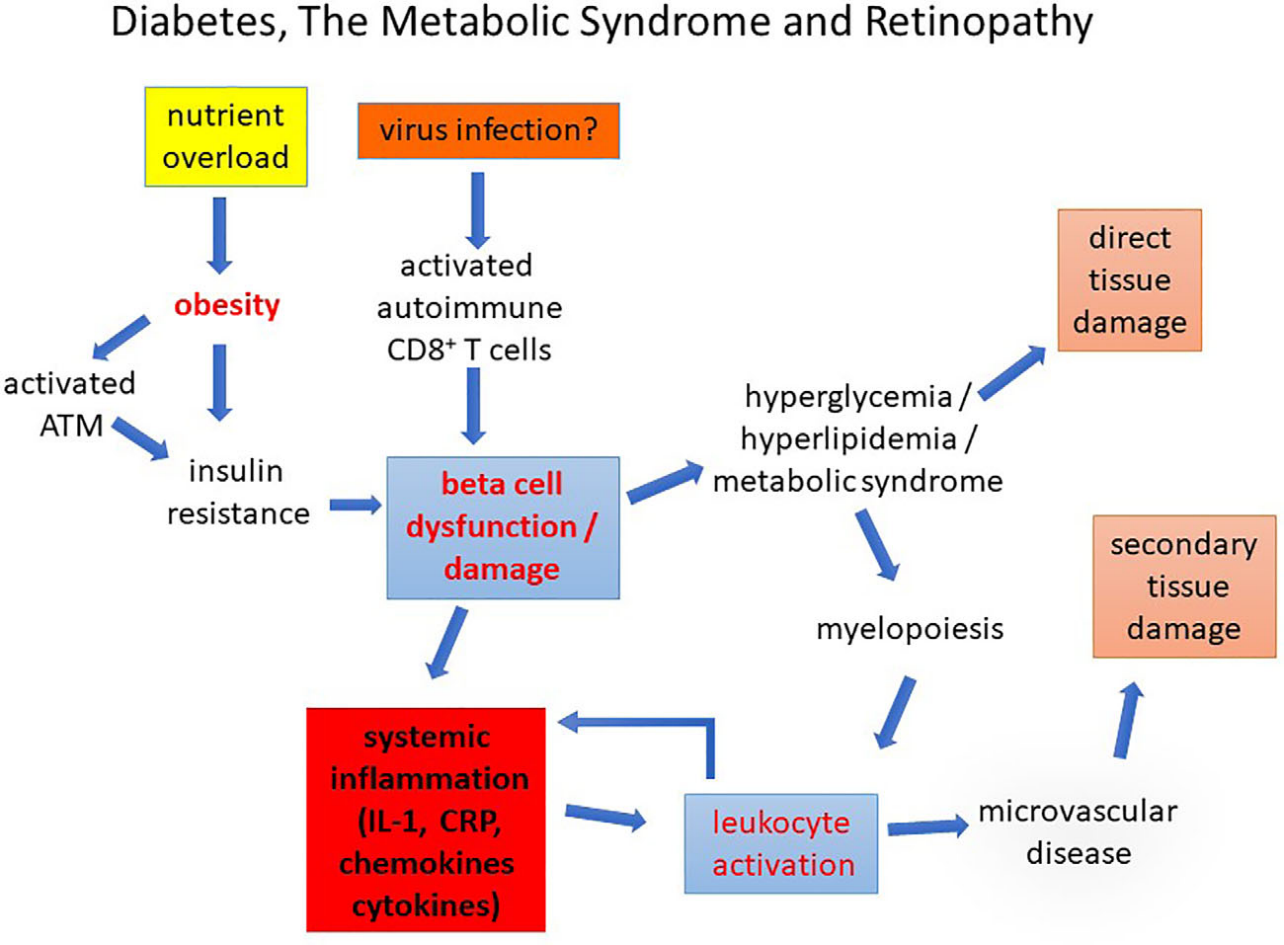
Diabetes, The Metabolic Syndrome and Retinopathy. Diabetes caused by nutrient overload or autoimmune disease leads to beta cell dysfunction/loss inducing a chronic pro-inflammatory state in which leukocyte activation promotes early microvascular disease. This causes secondary tissue damage (retinopathy, nephropathy, and peripheral neuropathy). Direct damage to the tissues by chronic glycolipid toxicity develops more slowly compounding the pathological changes.

In diabetes, the initial site of injury is the islet beta cell (discussed above) leading to cell death with local and systemic cytokine and chemokine release. This becomes a systemic challenge mediated by hyperglycemia in a self-perpetuating cycle which affects all tissues including the bone marrow (**Figure 9**). Thus, bone marrow-derived circulating leukocytes become persistently activated *in situ* and remain so as they enter the circulation and populate the tissues. This can occur as early as two weeks after the onset of hyperglycemia (145).

How does glucose activate mononuclear cells? Monocytes express GLUTs 1, 3, and 5 which they use to take up glucose by facilitated diffusion, i.e., in a concentration dependent manner (176–178). GLUT 1 in particular is upregulated in pro-inflammatory macrophages to support the upregulation of glycolytic metabolism (48, 179). Macrophages can also accumulate glucose against a concentration gradient in a glucose-deprived environment using sodium-glucose transporters (180, 181). However, GLUT 1 is the major transporter of glucose into monocyte/macrophages and is relatively insulin independent, i.e., uptake is determined by the extracellular glucose concentration. High levels of ambient glucose together with activation through exposure to tissue damage-release of cytokines such as IL-1β and TNF-α activate leukocytes to increase glucose uptake and metabolism, predominantly through aerobic glycolysis (Warburg effect) and increased hexokinase (Hx) activity. Hx converts glucose to glucose-6-phosphate in the initial stages of glycolysis and is itself a PAMP (see above, *Is Inflammation in Diabetes a Direct or an Indirect Cause of Complication*) which positively regulates leukocyte activation (84) while the glucose sensor pyruvate kinase M2 (PKM2) (see **Figure 3**), which normally combines with fructose 1,6-bis-phosphate to complete the production of phospho-enol pyruvate and pyruvate, is partially diverted to the inactive monomeric enzyme (182, 183). Monomeric PKM2 translocates to the nucleus where it activates hypoxia inducible factor-1α associated genes required for the production of VEGF (182, 183). HIF-1α is the driver of metabolic reprogramming in monocyte/macrophages as well as of angiogenic responses through VEGF (48, 179) (**Figure 3**).

Thus, activated circulating pro-inflammatory leukocytes trapped in the retinal microcirculation are not only agents of capillary non-perfusion and retinal ischemia but are sources of factors which can induce increased vascular permeability (VEGF/VPF) as well as induce local angiogenic responses (e.g., budding of m/a) (184). This adds to the already induced source of VEGF from activated retinal cells such as microglia and Müller cells (see above) but, because of its location inside the vessel, the activated, glucose-induced pro-inflammatory leukocyte can have direct access to the glycocalyx-depleted endothelium. Trapped leukocytes within the microaneurysm have been elegantly demonstrated by electron microscopy (185). As detailed above, in diabetes leukocytes express increased levels of activation molecules while the high glucose-exposed endothelium is likewise under stress and expresses the complementary surface adhesion molecules. Activated leukocytes in the presence of high glucose also promote inflammation by shunting of accumulating succinate intended to go through oxidative phosphorylation to production of HIF-1α (48) while excess succinate released extracellularly acts as a stimulant for further leukocyte activation and recruitment (186) (**Figure 2**).

As indicated above, glucose and its products comprise one set of metabolites which can induce leukocyte activation while increased lipid, dyslipidemia, and particularly “bad” lipids indirectly activate leukocytes *via* cytokine release as a result of dysregulated sphingolipid metabolism (79). As for DR, the retinopathy in HFD-diet fed mice appears to be linked to increased adhesion molecule expression (187). The common denominator in this pathology seems therefore to be chronic, but dysregulated, leukocyte activation which has widespread effects on tissues generally and the retina in particular (**Figure 9**).

## Leukocyte Activation in Diabetes—What Is the Mechanism?

Leukocyte activation in response to pathogen challenge or to tissue damage/injury follows a now well-defined path involving PAMP - PRR interactions, activation of distinct signaling pathways such as TLR - Type 1 interferon and C-Type lectin - inflammasome signaling as well as the cGAS-cGAMP-STING pathway, resulting in cytokine release with positive feedback amplification *via* central mediators such as IL-1β and TNF-α (188). How leukocytes become activated in diabetes and the MS is less clearly defined but probably follows a similar pathway (**Figure 2**). Beta-cell damage in T1D leads to IL-1 release which can initiate the process but later in T1D and, from the beginning, in T2D the inducers of inflammation are less clear. Hyperglycemia from beta cell loss or from nutrient overload, combined with microbiome-associated dysbiosis (100, 189) and low level endotoxemia may be sufficient to sustain low-level chronic leukocyte activation (**Figure 9**). Increased aerobic glycolysis and hexokinase activity (84) due to heightened glucose flux, intracellular accumulation and extracellular release of succinate with HIF-1α-gene activation and VEGF production (see above), are likely to contribute to overall low-level chronic leukocyte activation. Indeed, mTOR and HIF-1α mediated aerobic glycolysis underpin innate immune cell memory which may contribute to the ready activation of myeloid cells chronically exposed to metabolic stimuli. Most recently, it has been shown that circulating myeloid (monocyte/macrophage) cells in patients with diabetes, and in STZ-experimental mice, have elevated levels of pSTAT3, a cell signaling regulatory molecule while specific deletion of SOCS3 in the same cells (LysM-SOCS) leads to increased leukostasis and capillary drop out (190). Cytokine release, as well as damage-associated ATP release, entrains multiple signaling pathways including: (a) IL-1β/IL-1r signaling involving the Mydd88/Src/Asc-NLRP3/inflammasome pathway; (b) IL-6/IL-6r - MAPK; (c) TNFα/TNFαr and MAPK/NFκ B signaling; and (d) ATP/P2x7R - inflammasome pathway. Increased glycolysis by activated leukocytes also increases signaling through the succinate/succinate receptor and lactate is no longer seen as a bit player in this process. Lactate as well as being a source for mitochondrial respiration and energy *via* gluconeogenesis, can directly modify histone proteins and epigenetically modify gene activity (lactylation) (191).

However, metabolite/nutrient-associated leukocyte activation as in the MS and diabetes (both T1D and T2D) is different from leukocyte activation due to pathogen challenge. As indicated above, myelopoiesis is increased but there is a disconnect between HSC production and EPC mobilization such that vascular endothelial repair is defective. In addition the quality of macrophages in wounds is shifted more toward inflammatory macrophages than arginase^+^ wound healing macrophages such that wounds don’t heal well. These aspects of leukocyte activation have direct relevance to DR in which the retina attempts to restore tissue integrity at sites of retinal ischemia but may be unsuccessful (see above).

Several of these pathways are kinase dependent and regulated by phosphatases (192). The ubiquitous protein tyrosine phosphatase PTP1B has been shown to be integral to glucose and lipid metabolism in several tissues and control of energy and adiposity varies with the tissue location of PTP1B (193–197). Importantly, as indicated above macrophage and neutrophil specific PTP1B appears to drive a pro-inflammatory function since deletion of PTP1B specifically in myeloid cells protects against HFD and LPS-induced inflammation and endotoxemia (81, 198). A possible therapeutic intervention through modifying these signaling pathways might be possible.

Whatever the precise mechanism, chronic inflammation with activated leukocytes and circulating inflammatory mediators is clearly a significant feature of diabetes, obesity and the MS, is present from the onset of disease, and appears to be a direct cause of the characteristic microvascular disease leading to overt clinical symptoms such as DR (**Figure 9**).

## Diabetic Retinopathy—Is There a Role for Adaptive Immunity?

T1D is considered to be an autoimmune disease induced by viral infection (see above) while in T2D there is a marked reduction in AT Tregs, the homeostatic regulator of immune tolerance (61, 199). In all cases definitive autoantigens have not been identified but activation markers on systemic T cells are well recognized (see above). Autoimmunity as a driver of disease progression in DR has also been proposed. The constant, repetitive leukocyte-endothelial cell adhesive events occurring in the diabetic retina in time lead to significant retinal ischemia, retinal damage and release of damaged retinal proteins. Many tissue specific retinal proteins act as autoantigens although these mostly reside in the photoreceptors which are only affected once the disease is significantly advanced. Inner retinal damage to integral Müller cells, neurones and retinal glial cells also release autoantigenic peptides. Increased levels of various auto-antibodies have been detected in patients with DR (200–203) including one to hexokinase 1 (204), while, in one study, DR was more common in the absence of autoantibodies (e.g., to GAD). The evidence, therefore, for an autoimmune pathogenesis in DR is relatively limited (205). Interestingly, in the context of premature aging of endothelial cells in DR (see above), there also appears to be an accumulation of senescent T cells in the circulation of T2D patients with impaired glucose metabolism (206). Most recently, in a prospective pilot study, increased numbers of circulating neutrophilic leukocytes with a reduced number of T cells during the development and progression of DR was observed (207). At this stage, it is difficult to define the role of adaptive immunity in DR.

However, the possibility remains. Circulating T cells participate in retinal leukostasis creating the potential for T cell activation by bone marrow derived DC through presentation of retinal peptides. Circulating pre-DC comprise a component of CD11b^+^ myeloid cells implicated in STZ-mouse DR-associated leukostasis and so all the ingredients for an autoimmune component to DR are present in the pathological retinal milieu. We have also recently shown that inhibition of PTP1B in dendritic cells inhibits T cell activation (208) and so the same mechanism that inhibits HFD-induced obesity may also be active in diabetes-linked adaptive immunity. Overall, however, it is likely that activation of innate immunity begins the process of DR while any role for adaptive immunity is likely to be in the further progression of the disease.

## Preventing and Treating the Microvasculopathy of Obesity/the MS/and Diabetes

Many strategies have been proposed for prevention and treatment of the complications of diabetes. In the case of DR, most treatments are delivered when the condition is sight threatening. PDR and severe non-PDR are treated by retinal laser photocoagulation therapy (LPT) based on data from the Early Treatment for Diabetic Retinopathy Study (ETDRS) and, since its introduction, LPT has become the standard treatment, although the original evidence is not entirely robust [reviewed in ref. (209)]. LPT is destructive and areas of retinal damage (atrophy) may increase long after end of treatment (210, 211). Its mechanism of action is unclear. Reduction in VEGF release from oxygen starved cells in the retina is the accepted explanation for the effectivity of LPT. This could include retinal inflammatory cells such as macrophages as well as parenchymal cells since LPT induces indiscriminate and extensive cell death. Randomized controlled clinical trials (RCT’s) have shown that inhibiting VEGF production by using intravitreal anti-VEGF agents is also effective, but not entirely so, particularly for macular edema [reviewed in (212–214)], indicating that there are other factors at play in the tissue fluid accumulation. In addition, the same studies showed that intravitreal steroid injection may be equally effective as anti-VEGF therapy in the short term and have a more durable long term effect. A combination of both treatment modalities (LPT in association of anti-VEGF) seems to be the most beneficial approach for many patients with advanced DR.

Few RCT have explored preventive therapies for DR, i.e., clinical trials initiated at time of diagnosis of diabetes. The first prospective study of intensive glycemic control (IGC) vs. conventional therapy clearly demonstrated a beneficial effect of strict blood glucose control in preventing or delaying onset of DR in T1D (215–217). Furthermore, early initiation of strict glycemic control has had long lasting beneficial effects (218). A landmark study in T2D, the United Kingdom Prospective Diabetes Study (UKPDS) also clearly showed that IGC reduced the incidence of DR and sight loss (219) and later re-analysis of this and other studies confirmed this finding. However, in both the DCCT and the UKPDS the risk of hypoglycemic attacks has prevented IGC from being uniformly and strictly applied (220–222).

A search for a safer systemic approach to preventing or delaying development of complications has re-examined the benefits of the lipid-lowering drug fenofibrate (223, 224), but the, albeit uncommon, side-effects have also prevented its widespread use. Experience with the liberally prescribed statins has also been examined as a possible means of eliminating the hyperlipidemic risk factor for DR, and there is some evidence from RCT’s (225–227) but a critical analysis of published data suggests that the quality and reporting of the data may be flawed (228). Experimentally, rosuvastatin has been shown to inhibit leukostasis in which the effect appeared to act on the endothelium rather than the leukocytes, thus pointing to a primary metabolic defect in the endothelium initiating leukostasis (145).

However, both fenofibrate and statins have more than a lipid lowering effect. In particular, there is considerable evidence that anti-inflammatory effects of statins are its a major mechanism of action whether it is by acting on the endothelium or directly on inflammatory cells (229–231). Directly inhibiting inflammation in the treatment of diabetes has a long history, even to the early use of salicylates [reviewed in (232)], and has been shown to have some effect. However, for control of DR, such treatments have been introduced too late: as outlined in this review, pathologies like macular edema are late, if not very late, events and permanent structural damage has already been done by the time vision is affected. Therefore, the likelihood of reversing disease is small, but does happen. Patients with mild background retinopathy not involving the macula have had chronic inflammatory disease for many years and early treatment before vision is threatened is the only way blindness can be stopped. The complications of diabetes are initiated from the onset of disease and by controlling the triggers of inflammation such as hyperglycemia and dyslipidemia loss of vision can be prevented as has already been shown (219). The possibility of direct inhibition of IL-1 using the monoclonal anti–IL-1 antibody gevokizumab has been proposed but indications and timing of therapy are not decided (233). A second anti-IL1 moAb, canakinumab has been proposed for inflammation control in cardiovascular disease but not so far for diabetes (97, 98). In the recent CANTOS trial no benefit for new onset diabetes either in prevention or management was seen with canakinumab (234). However, it may be that it will be very difficult to design an RCT which could demonstrate an effect of a broad-spectrum or molecule-specific anti-inflammatory strategy while the fundamental metabolic defect affecting all cells, including inflammatory cells, remains.

## Conclusion

Our understanding of the role of inflammation in diabetic retinopathy is taking shape. While hyperglycemic glucose toxicity affecting retinal cells and retinal vessel endothelium undoubtedly induces retinal neurodegeneration and vasculopathy respectively, these changes almost certainly are not primary events. Instead, in T1D pancreatic inflammation and beta cell damage/loss set a proinflammatory scene from the outset while in T2D, AT/obesity-associated pre-diabetic inflammation begets IR and hyperglycemia which amplifies immune cell activation and continues the cycle of chronic inflammation (**Figure 9**). Retinal vessel endothelial changes, due in part to premature senescence, become an attractant for leukocytes to adhere, probably as part of an endothelial repair process. However, this fails to deliver, in part because the HSC-niche in the bone marrow shifts from generating physiologically reparative EPCs to pro-inflammatory macrophage-like progenitors. Despite an overall increase in myelopoiesis, this fails to restore vascular integrity, and instead promotes vascular occlusions and increasingly large areas of ischemia especially in the retina. These changes may be observed even before hyperglycemia as in the HFD mouse model and in HR in humans and are a measure of the direct effect of overall immune cell activation and immunometabolism underpinning the MS and diabetes.

## Author Contributions

JVF researched and wrote the manuscript. LK wrote the manuscript and provided figures. MD wrote the manuscript and revised the figures. All authors contributed to the article and approved the submitted version.

## Funding

This work was supported by grants from the Development Trust of the University of Aberdeen: grant number: RG14251. The authors acknowledge the continuous support from Saving Sight in Grampian (Charity No.SC002938), and support from Fighting for Sight Aberdeen (Charity No.SC033004) for open access publishing fee.

## Conflict of Interest

The authors declare that the research was conducted in the absence of any commercial or financial relationships that could be construed as a potential conflict of interest.
